# An Evaluation of the Role of Simulation Training for Teaching Surgical Skills in Sub-Saharan Africa

**DOI:** 10.1007/s00268-017-4261-7

**Published:** 2017-10-11

**Authors:** Nicholas J. Campain, Mithun Kailavasan, Mumba Chalwe, Aberra A. Gobeze, Getaneh Teferi, Robert Lane, Chandra Shekhar Biyani

**Affiliations:** 1 Royal Devon and Exeter NHS Foundation Trust, Exeter, UK; 2 Royal Derby Hospital, Derby, UK; 3 University Teaching Hospital, Lusaka, Zambia; 4 Hawassa University and Referral Hospital, Hawassa, Ethiopia; 5 The Association of Surgeons of Great Britain and Ireland, London, UK; 6 Department of Urology, St James’s University Hospital, Leeds Teaching Hospitals NHS Trust, Beckett Street, Leeds, LS9 7TF UK

## Abstract

**Background:**

An estimated 5 billion people worldwide lack access to any surgical care, whilst surgical conditions account for 11–30% of the global burden of disease. Maximizing the effectiveness of surgical training is imperative to improve access to safe and essential surgical care on a global scale. Innovative methods of surgical training have been used in sub-Saharan Africa to attempt to improve the efficiency of training healthcare workers in surgery. Simulation training may have an important role in up-scaling and improving the efficiency of surgical training and has been widely used in SSA. Though not intended to be a systematic review, the role of simulation for teaching surgical skills in Sub-Saharan Africa was reviewed to assess the evidence for use and outcomes.

**Methods:**

A systematic search strategy was used to retrieve relevant studies from electronic databases PubMed, Ovid, Medline for pertinent articles published until August 2016. Studies that reported the use of simulation-based training for surgery in Africa were included.

**Results:**

In all, 19 articles were included. A variety of innovative surgical training methods using simulation techniques were identified. Few studies reported any outcome data. Compared to the volume of surgical training initiatives that are known to take place in SSA, there is very limited good quality published evidence for the use of simulation training in this context.

**Conclusions:**

Simulation training presents an excellent modality to enhance and improve both volume and access to high quality surgical skills training, alongside other learning domains. There is a desperate need to meticulously evaluate the appropriateness and effectiveness of simulation training in SSA, where simulation training could have a large potential beneficial impact. Training programs should attempt to assess and report learner outcomes.

## Introduction

An estimated 5 billion people worldwide lack access to any surgical care [1] and surgical conditions account for 11–30% of the global burden of disease [2]. Sub-Saharan Africa (SSA), the region with the most severe health disparities, demonstrates a desperate lack of healthcare providers to deliver surgical care, particularly in rural areas. For example, in Malawi, there is one urological surgeon for a population of 16 million people, compared to the UK where the ratio is 1 urologist to 65,769 people [3]. The World Health Organisation (WHO) have recommended ‘task shifting’ by training ‘non-physician clinicians’, such as medical officers, to perform some surgical procedures in an attempt to address this shortage [4, 5]. It is now recognised as a global public health priority to improve access for patients to essential and safe surgical care. Newer educational pedagogies, such as simulation training, may have an important role in up-scaling and improving the efficiency of surgical training in resource-poor countries. A survey by Ahmad and Mishra has reported disparities in access for African trainees to minimal access surgery training facilities [6].

Simulation training in surgical education has been studied extensively; however, there is very limited evaluation of the use of simulation training for surgical skills in SSA, the region where there could arguably be the greatest benefit.

This critical review will evaluate the use and types of surgical simulation training interventions in SSA and assess the quality of evidence used in their assessment. Challenges of simulation training in this environment will be considered, and applicability and potential developments for the future will be discussed.

## Methods

A systematic search strategy was used to retrieve relevant studies from electronic databases PubMed (http://www.pubmed.gov) and Ovid, MEDLINE (https://ovidsp.tx.ovid.com) for pertinent articles published until August 2016. Core texts in medical education [7, 8] were consulted to provide an overview of the evidence for simulation training in general and the applications for surgical skills training. The following search terms were used to determine a P.I.C.O. framework question: ‘simulation’, ‘training’, ‘surgery’, ‘surgical skills’, ‘Africa’, ‘resource-poor’ and ‘low-income’. A manual hand search was also performed to identify all eligible studies. Inclusion of articles was determined by independent assessment by two reviewers. Resulting titles and abstracts were analysed for relevance and inclusion in the review. Studies that reported the use of simulation-based training for surgery in Africa were included.

**Fig. 1:**
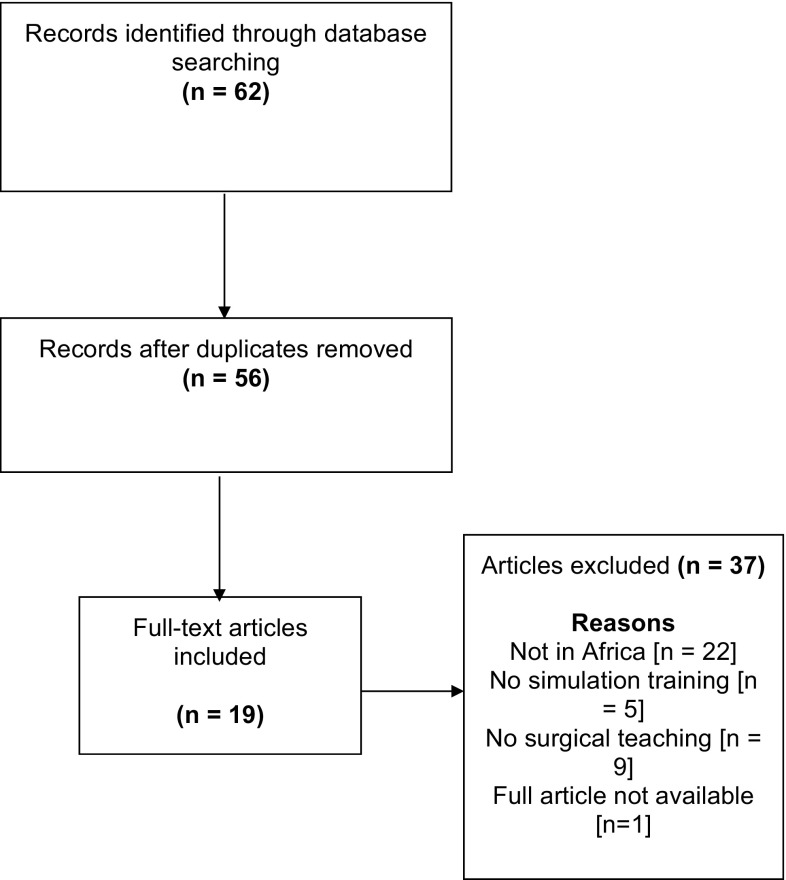
PRISMA flow diagram

In total, thirty-six articles were excluded from this web-based search (Fig. 1). Contemporary studies published in higher quality medical education journals were prioritised and references assessed to identify other relevant articles. Due to a limited number of studies in this research area, all relevant articles were included and the search terms were then broadened to include ‘medical education’, ‘clinical education’, ‘obstetrics’, ‘paediatrics’ and other medical specialties, in order to identify other articles describing the use of simulation in SSA. Particular attention was paid to articles detailing qualitative methodologies. No specific literature or systematic reviews regarding surgical simulation training in SSA were identified.

## Results

There is very limited published evidence for the use of simulation training in SSA (Table 1). Bench top simulation models used in high-income countries for teaching generic surgical skills have been used in SSA, and shown to be feasible and demonstrate marginal improvements using validated global-rating scales and skill-specific checklists [14]. Simulation models using low-cost, locally sourced models have also been used during training courses to attempt to address gaps in surgical skills training [15, 26].

**Table 1:** All designs are single group cohort (non-randomised, papers included)

Author	Year	Primary country/continent of simulation teaching	Primary specialty	Primary target group	Type of simulation	No. of study participants
Andreatta et al. [9]	2011	Ghana	Obs and Gynae	Nurse midwives	Surgical skills	111
Beard et al. [10]	2014	Tanzania	General surgery	Surgical residents	Surgical skills	14
Bergman et al. [11]	2008	Tanzania	Trauma surgery	Combined physicians and nurses	Trauma resuscitation	20
Biyani et al. [12]	2013	Ethiopia	Urology	Surgical residents	Surgical Skills	N/A
Campain et al. [13]	2016	Africa	Urology	Surgical residents	Surgical skills	74
Dorman et al. [14]	2009	Ethiopia	General surgery	Surgical residents	Surgical skills	19
Dreyer et al. [15]	2014	Zambia	General surgery	Surgical residents	Surgical skills	24
Jacobs et al. [16]	2010	Ghana	General surgery	Surgical residents	Surgical skills	71
Kigozi et al. [17]	2011	Uganda	Urology	Surgical residents	Anatomical validation	N/A
Livingston et al. [18]	2014	Rwanda	General surgery	Surgical residents	Surgical skills	N/A
Long et al. [19]	2014	Kenya	General surgery	Surgical residents	Surgical skills	10
Mikrogianakis et al. [20]	2011	Botswana	Orthopaedics	Residents	Telesimulation	22
Nelissen et al. [21]	2014	Tanzania	Obs and Gynae	Combined physicians and nurses	Surgical education	89
Okrainec et al. [22]	2009	Botswana	General surgery	Surgical residents	Surgical skills	20
Okrainec et al. [23]	2010	Botswana	General Surgery	Surgical consultants	Telesimulation	16
Perosky et al. [24]	2011	Ghana	Obs and Gynae	Nurse midwives	Anatomical validation	10
Saad AM [25]	1991	Sudan	ENT	Medical students	Verbal simulation	196
Taché et al. [26]	2009	Tanzania	General surgery	Medical students	Surgical skills	36
Tansley et al. [27]	2016	Rwanda	General surgery	Surgical residents	Surgical skills	26

Procedure-specific models have been assessed. A wooden penile model has been described for teaching male circumcision in Uganda but did not seek any feedback from trainees [17]. A reusable, low-cost, high fidelity simulated model has recently been developed and used to teach circumcision to 74 trainees from 7 African countries. To establish content validity, feedback from trainers was analysed (appearance and realism were rated as ‘very good’ or ‘excellent’ in 94 and 63%, respectively). Face validity was assessed by seeking feedback from trainees, who rated the model as either ‘very good’ or ‘excellent’ for appearance and realism of suturing in 60 and 71%, respectively [13].

Higher level surgical skills, such as laparoscopic skills training, have been evaluated in a small number of studies in SSA. Laparoscopic simulation training assessed in one small study of 10 Kenyan surgical residents demonstrated improvements in performing a simulated procedural skill with a box trainer model [19]. More recently, a low-cost box trainer has been effective in improving simulated technical skills using a validated system for training and evaluation of laparoscopic skills [10]. A three-day course in Botswana also demonstrated improvements in knowledge and technical skills for a ‘Fundamentals of Laparoscopic Surgery’ (FLS) course using a simulator model [22]. Teaching FLS via telesimulation can be successful, with a 100% pass rate in a telesimulation group compared to 38% in a self-practice group [23]. Other box-simulator models, such as the Bristol transurethral resection of the prostate simulator, have been used in the development of endoscopic urological services in Ethiopia, but did not report any specific learner outcomes [12].

The use of simulation models and scenarios amongst 20 participants, in a trauma team training programme in Tanzania, have shown improvements in knowledge and demonstrated good construct validity of a questionnaire. A novel trauma team assessment tool was also developed [11]. Other trauma management courses that utilise simulation training are active in SSA but have not reported any outcomes [28].

In the paediatrics literature, ‘telesimulation’ using a remote internet link between Canada and Botswana has shown improvements in physicians’ knowledge and self-reported confidence in performing a procedural skill (insertion of an intraosseous needle) [20]. In another low-resource setting in Ghana, improvements in knowledge (operative steps) and self-reported confidence were demonstrated in a small study of 12 obstetric students compared to a control group [9].

## Discussion

Simulation ‘is a technique to replace or amplify real-patient experiences with guided experiences, artificially contrived, that evokes or replicates substantial aspects of the real world in a fully interactive manner’ [29]. Simulation, as an educational strategy, provides learning opportunities that are both immersive and experiential. It is broadly used in three domains within healthcare: to teach technical or procedural skills; to assess performance of clinical skills; and in complex team training scenarios [30]. To enable proficiency at a procedural skill, simulation training must ideally incorporate other domains of learning, such as communication skills, as performing a procedure in the ‘real’ world of clinical scenarios is complex and requires more than just the technical ability. As stated by Gaba [31] ‘simulation, when integrated appropriately into learning and competence testing, plays an important role in acquiring the critical and reflective thinking skills needed to provide competent, safe patient care’. In 1956 Bloom first described a classification system of different learning objectives for students [32], comprising of three ‘domains’: cognitive, affective and pyschomotor. Teaching surgical skills with simulation falls predominantly within the psychomotor domain; however, one aim of Bloom’s taxonomy is to encourage educators to also consider the other domains. Applying this principle should therefore be considered in any simulation teaching in order to create a holistic teaching environment and maximise learning opportunities.

This review has focus predominantly on the teaching of simulated surgical skills. This ranges from the use of low-fidelity models, animal or cadaveric tissue and high fidelity video simulators. Educational theories underpinning simulation allow the learner to gain a concrete experience as described by Kolb’s cyclical model of experiential learning (although ‘artificial’) in a safe environment, without the ethical dilemma of using real patients [33].

This can promote effective learning through active learner engagement, repetitive practice, the ability to vary difficulty and clinical complexity, as well as diagnostic performance measurement and intra-experience feedback [34, 35].

A systematic review, in 2008, attempted to determine whether skills acquired by simulation training could be transferred to the operative setting. Ten randomised controlled trials (RCTs) were included and the study concluded that skills acquired did appear to be transferable, by assessing parameters such as performance time and ability to complete the procedure [36]. However, methodological weakness was noted, as, in most of the studies, trainees received simulation training in addition to normal training and the strength of the conclusion was limited by the inclusion of studies of variable quality.

More recently, in 2014, a systematic review of 16 RCTs, involving 309 participants, found that the simulation literature consistently showed benefits in terms of operative time and performance scores [37]. However, it was again acknowledged that further study is required before it can be concluded that simulation skills are directly transferable. One smaller observational cohort study, assessing simulation for performing a procedural skill (insertion of a central venous catheter), has demonstrated improved efficiency and a reduction in complications relating to real-life patient care [38]; however, this was again limited by methodological flaws. Assessing competency to perform a task is complex and demonstrating that simulation training has direct benefits on clinical outcomes is difficult. This must be considered in the context of simulation training in SSA as measures to assess the effects on real-life patient outcomes are yet to be fully evaluated.

Simulation training has predominantly been studied in the context of Western educational models in high-income countries. The generic challenges of simulation training will be similarly applicable in SSA. There remains debate regarding the transferability of skills learnt during simulation training to the real-life operative setting [34, 39] and this has not been investigated in the low-resource setting of SSA. The educational context is important in simulation training, as it must be realistic, patient-focused, structured and grounded in an authentic clinical context [40].

In addition to the general challenges associated with simulation training, there are specific issues to simulation training in SSA. Barriers to adoption of simulation training include cost and resource requirements, such as teaching faculty and requirement for an appropriate learning environment [41]. These challenges may be even greater in SSA and can be broadly categorised as either logistical (relating to the delivery of simulation training) or methodological (the educational context).

In SSA, finding an appropriate teaching environment or space can be difficult. Collaborations to develop dedicated simulation centres exist [18], but these rely on external sources of funding. Simulation training may have limited applicability in most institutions due to these resource constraints, but low-cost models have been used successfully in some centres [26].

The availability, preparation and storage of cadaveric or animal material for surgical simulation models can also be challenging. Additionally, running a course in an inadequately sized and poorly ventilated hot room can lead to a sub-optimal learning environment. Cultural acceptability must also be considered, for example the use of porcine material for simulation models in a Muslim country.

Technological restraints can hamper effectiveness of simulation models due to problems with sporadic electrical power supply or internet connectivity in SSA. Telesimulation links have been demonstrated [20, 23] but applicability and scalability to other centres may be limited.

Most simulation programmes described in SSA have been supported via overseas organisations, often with provision of short-term overseas trainers [12, 14, 15, 22, 26]. Organising local faculty support in countries with an already limited number of surgeons, combined with weak transport infrastructure and large distances can make it challenging to deliver teaching interventions. This may also lead to dependence on visiting faculty unless local educators are simultaneously trained [15]. Communication and language issues can also arise.

Surgeons in SSA may require a different skill set to deal with different surgical pathologies compared to surgeons in high-income countries, owing to a different nature and frequency of surgical pathology [42]. Designing learning objectives that are responsive to locally defined needs and teaching appropriate skills is imperative, yet unfortunately there are examples of interventions that do not adhere to this basic educational principle. Simulation training has been used to train surgeons who are then subsequently unable to perform learned procedures due to inability to access equipment in their own institutions [43].

There are cultural implications for newer methods in medical training, and studies carried out in multi-cultural institutions [44] and in Asian medical schools [45] have shown different learner attributes following the introduction of problem-based learning. African medical schools have generally not embraced innovations in educational pedagogies and lack trained medical educators [46]. Newer forms of teaching, such as simulation training, may not be as effective or appropriate in this setting, and studies are required to assess the cultural acceptability. The effect of hierarchy, inter-professional learning and the dynamics of diverse cultural groups, particularly in team-based simulation scenarios, require further investigation in the SSA setting.

Simulation for surgical skills training in high-income countries has been extensively studied, and two systematic reviews of RCTs have concluded that skills learnt are transferable to the operative setting, with measured improvements in some parameters such as operative time [38, 40]. In SSA, there is very limited evidence of the use of simulation. There are a very limited number of published studies and publication bias may be present within this limited literature base. There are no RCTs and most studies have small numbers of participants. Several studies originate from one urban institution in Tanzania [10, 26], so results may not be generalisable to other locations within SSA. The quality of available evidence is generally poor, with notable limitations to methodologies used. Some studies did not use a control group for comparison of teaching interventions, as it was felt unethical to not offer educational interventions to surgeons in resource-poor settings [10]. Whilst commendable and understandable, this does limit the strength of any conclusions drawn.

Specific simulation models have been validated in high-income countries [47] but there are few examples of either new or existing models being validated for use in the different and diverse educational setting of SSA. Studies which have attempted to assess validity in SSA have only sought self-reported feedback from participants [13, 14] and so no conclusions on transferability to real world operative practice can be drawn.

Some studies in SSA have demonstrated short-term improvements in knowledge and performance in technical skills [10, 19, 22, 26], but almost universally lack any longitudinal data to assess whether these were retained. Only one study has assessed self-reported performance at 6 months following a course which included both lecture based and simulated procedural teaching methods, with 90% participants reporting increased ability to manage surgical emergencies [15].

Simulation training in SSA is much more prevalent than the published literature suggests. There are 66 registered surgical organisations within the region covered by the College of Surgeons of East, Central and Southern Africa and many smaller non-governmental organisations that may be involved with surgical training courses. Many of these run courses or educational activities that utilise some form of simulation training. There is also a dedicated surgical skills centre in Nairobi, Kenya, supported by the pharmaceutical industry, which regularly runs simulation workshops. This large volume of unpublished simulation activity is encouraging, but also represents missed opportunities to analyse and validate the effectiveness of educational methodologies.

Low or medium fidelity models may work best in resource-limited settings [7], such as SSA, and cheap simulated models have been described [13, 17, 26]. A low-cost simulator for bimanual compression training for the management of postpartum haemorrhage has been used in Ghana to reduce maternal mortality [24]. However, finance resources for education in SSA are low and it is not clear what educational interventions for learning surgical skills are most cost-effective.

In Gambia, for community healthcare workers, the use of a standardised patient was effective in developing an educational programme to teach management of sexually transmitted diseases [48].

Recommendations

1. Simulation for teaching surgical skills is feasible and widely used in SSA, but there is very limited published data to support the use specifically in this setting. Research in this area should be promoted in order to improve effectiveness of educational interventions.
2. Teaching programmes that utilise simulation should attempt to improve methodological study design, irrespective of geographical location. Research in this setting is complex; however, it is part of ethical educational practice to assess effectiveness and translation to real-life clinical outcomes, particularly in resource-poor settings. Greater efforts to understand longitudinal benefits of simulation training and retention of knowledge are a priority.
3. Simulation is valued by learners to gain experience to practice surgical skills and this may have even greater applicability in resource-limited settings such as SSA [26]. Interventions in SSA should also focus on other educational outcomes, not just psychomotor domains, to understand the learning and cultural environment more fully.
4. Technological improvements offer exciting benefits for remote training methods in SSA, such as telesimulation and mentoring [22, 23]. Further evaluation of suitability and barriers to implementation is required.
5. Further understanding of the simulation pedagogy in general may enable deconstruction of the key elements that improve ability to perform technical skills. Exciting areas for future study include evaluation of ‘mental practice’ to enhance performance of tasks which has been shown to be effective in an RCT [49]. This should be a research priority, as a low-cost technique with enormous potential transferability to SSA.

## Conclusions

Global surgical training is a public health priority. Simulation teaching presents an excellent modality to enhance and improve both volume and access to high-quality surgical skills training, alongside other learning domains. The applicability may be greatest in resource-poor settings such as SSA, however many research questions remain unanswered. Accepting the methodological challenges and limitations of simulation training in general, there is a desperate need to rigorously evaluate the suitability and effectiveness of simulation training in SSA. Several areas offer potential promise, such as the use of telesimulation and newer low-cost high fidelity models.
